# Parental conflict and adolescents’ socially adverse emotions: the mediating role of family functioning

**DOI:** 10.3389/fpsyg.2024.1387698

**Published:** 2024-10-09

**Authors:** Rong Kong, Ruihua Chen, Lingling Meng

**Affiliations:** ^1^Key Research Base of Humanities and Social Sciences of the Ministry of Education, Academy of Psychology and Behavior, Tianjin Normal University, Tianjin, China; ^2^Faculty of Psychology, Tianjin Normal University, Tianjin, China; ^3^Center of Psychological Education and Counseling, Taiyuan Institute of Technology, Taiyuan, China; ^4^School of Marxism, Beijing Polytechnic College, Beijing, China; ^5^Economy and Technology Department, Shanxi Trade School, Taiyuan, China

**Keywords:** parental conflict, family functioning, shyness, loneliness, adolescents, mediation

## Abstract

**Objective:**

To examine the process of how parental conflict and family functioning influence adolescents’ socially adverse emotions (shyness and loneliness).

**Methods:**

Stratified cluster sampling was used to conduct a questionnaire survey among 1,100 junior high school students from three junior high schools in Beijing, Chongqing, and Shijiazhuang, China.

**Results:**

(1) The overall experience of adolescents’ socially adverse emotions was at the moderate level; boys’ experience of shyness and loneliness was significantly higher than that of girls; the experience of shyness and loneliness in the second grade was significantly higher than that in the first grade; (2) Parental conflict was significantly negatively correlated with family functioning and significantly positively correlated with adolescents’ socially adverse emotions, while family functioning was significantly negatively correlated with adolescents’ socially adverse emotions; (3) Family functioning partially mediates the relationship between parental conflict and adolescents’ shyness and completely mediates the relationship between parental conflict and adolescents’ loneliness.

**Conclusion:**

Compared to adolescents’ shyness, family functioning plays a more important mediating role in the relationship between parental conflict and adolescents’ loneliness.

## 1 Introduction

As one of the most common mental disorders, social anxiety refers to the emotional experience of nervousness and anxiety generated by individuals who worry about being negatively evaluated when talking with others or participating in social activities (Beidel et al., 1995; de Ponti et al., 2024). The Fifth edition of the diagnostic and statistical manual of mental disorders (DSM-V) classifies social anxiety disorder as an “anxiety disorder” (Pittelkow et al., 2021). People with social anxiety disorder fear scrutiny, fear saying or doing the wrong thing, and feel uncomfortable in unfamiliar social situations. They will try their best to avoid social interaction, do not take the initiative to interact with others, and avoid speaking in public. At the same time, they will endure strong emotional experiences in unavoidable interpersonal interactions (Cox et al., 2004). A survey conducted in seven countries around the world found that the prevalence of social anxiety among adolescent teenagers ranges from 23 to 58% (Jefferies and Ungar, 2020). Extensive research has been undertaken to clarify its factors, and researchers have made several suggestions on how to cope with it (de Ponti et al., 2024). However, little is known about the emotions that go along with it such as shyness and loneliness. It has been indicated by some researchers that adolescents with high levels of shyness and loneliness have difficulty in establishing social relationships (Ime et al., 2024). In other words, shyness and loneliness are two important indicators of adolescents’ socially adverse emotions.

Shyness is an individual’s tendency toward experiencing fear, inhibition and awkwardness in situations in the presence of a stranger or when perceiving social evaluation (Hipson and Coplan, 2018). A large number of previous studies have shown that shyness affects adolescents’ social and emotional adjustment. For example, shyness positively and significantly predicts adolescents’ social anxiety (Ime et al., 2024; Zeytinoglu et al., 2022), depressive symptoms (Wang et al., 2022), academic procrastination (Sun et al., 2024), internet gaming disorder (Wang et al., 2022), and substance use (Lemyre et al., 2019). Shyness also plays a role in adolescents’ friendship selection (Yang et al., 2021) and has a strong association with peer relations (Liu et al., 2019).

Loneliness describes the negative emotional experience that occurs when there is a large gap between the desired social relationships and the actual situation in terms of quality (such as satisfaction with relationships or perceived social acceptance) or quantity (such as frequency of socializing and number of friends) (Maftei et al., 2024). The harm of loneliness to adolescents has been supported by a large body of empirical research. In the study conducted by Pecora et al. (2024), loneliness was found to be negatively associated with adolescents’ mental health and sleep quality. Besides, long-term loneliness not only positively predicted problematic social networking sites use but also positively predicted social anxiety and negatively predicted social self-efficacy (Sun et al., 2024). The strong association between loneliness and adolescents’ problematic smartphone use has also been proved in a recent study (Zhao et al., 2024).

Parental conflict refers to an openly hostile relationship between married, separated, or divorced parents, which is considered a destructive conflict that may threaten an individual’s emotional sense of security (Cummings and Miller-Graff, 2015; Goering and Mrug, 2023). Some studies found that high frequency and intensity of parental conflict in the family system has a negative impact on children’s mental health (Deng and Li, 2024; Espinoza et al., 2024; Giallo et al., 2022), since it causes both parties to focus on their conflict, reducing sensitivity to their children’s needs (Deng and Li, 2024). Therefore, parental conflict may be a potential destructive factor that leads to children’s feelings of loneliness and shyness. On the other hand, according to the “person–situation interaction theory,” parental conflict is one of the remote environmental factors that affect adolescents’ psychological adjustment. This theory believes that the far-end environment in the family system often affects an individual’s emotion by affecting the function of the proximal environment (Kroencke et al., 2023). Therefore, parental conflict may indirectly affect children’s loneliness and shyness through other variables (Giallo et al., 2022).

**Figure 1:**
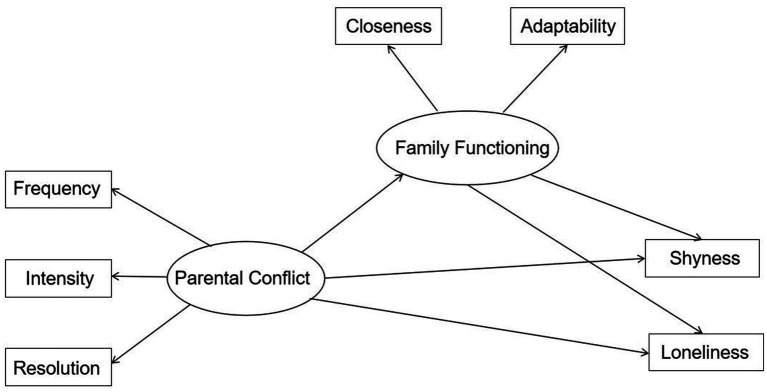
The hypothesized conceptual model.

Family functioning reflects the emotional connection between family members and the ability of the family to solve problems together (Bandura et al., 2011; Monteleone et al., 2024). Some studies found that family functioning is a factor affecting adolescents’ emotion (Monteleone et al., 2024) such as loneliness experience (Qian et al., 2022). Based on the definition of family functioning, we assume that, compared to parental conflict, family functioning should be a proximal factor, which has been supported by some empirical studies. In these studies, the relationship between husband and wife is an important factor affecting a number of dimensions of family functioning (Anant and Raguram, 2005; Ronaghan et al., 2024), and a poor relationship between husband and wife makes family functioning in an adverse state. Therefore, it can be speculated that conflict between parents may lead to family dysfunction, which will further affect the psychological development of teenagers. The first question this study focused on was whether parental conflict has an impact on adolescents’ shyness and loneliness. The second question we focused on was whether family functioning has a mediating role in the relationship between parental conflict and adolescents’ shyness and loneliness. Based on the literature review, our hypotheses were as follows: (a) there was a significant positive correlation between parental conflict and adolescents’ shyness and loneliness, i.e., the higher the level of parental conflict, the more likely adolescents would experience more shyness and loneliness; and (b) family functioning played a mediating role in the relationship between parental conflict and adolescents’ shyness and loneliness (see Figure 1).

## 2 Methods

### 2.1 Participants and procedures

An initial sample of 1,100 junior middle school students in grades 7 and 9 were recruited from three junior middle schools in Beijing, Chongqing, and Shijiazhuang, three cities in China, 62 of whom were excluded from the data analysis due to incomplete data (attrition rate = 5.64%). The final sample consisted of 1,038 students (480 boys and 554 girls, and four gender missing). The proportions of each grade were 46.1, 41.6, and 12.2%, respectively. All participation was voluntary. Prior to data collection, the participating schools, the student participants and their parents were informed about the research’s objectives, confidentiality and anonymity. According to the information provided by the participating students, 96.43% of them have intact families, i.e., their parents are not separated or divorced. During data collection, assessments of the study variables (i.e., family functioning, parental conflict, shyness, and loneliness) were administered to the participants using standard instructions in a group setting with ~40–50 participants in regular classrooms. The assessment procedure took ~50 min to complete.

### 2.2 Instruments

#### 2.2.1 Family functioning

Family functioning was measured using the 30-item adapted Chinese version of the Family Functioning Scale (Fei et al., 1991). Each item was answered on a scale ranging from 1 (totally disagree) to 5 (fully agree), with higher scores indicating a higher level of better family functioning. This scale consists of two dimensions: family closeness (e.g., Family members feel emotionally close to each other) and family adaptability (e.g., In our family, we try new ways to solve problems). We used the mean score for all the scale items as a total score. Cronbach’s *α* in the present study of each dimension and the total scale were 0.85, 0.87, and 0.93, respectively. This scale is one of the most used scales measuring family functioning, including among adolescents, showing good psychometric properties in previous studies (Vilca et al., 2024).

#### 2.2.2 Parental conflict

Parental conflict was measured using the conflict characteristics subscale from the Chinese version of Children’s Perception of Inter-parental Conflict Scale (Grych et al., 1995). This subscale has a total of 19 items, which are composed of the three dimensions of conflict frequency (e.g., I often see my parents arguing), conflict intensity (e.g., my parents have broken or thrown things during an argument), and conflict resolution (e.g., even after my parents stop arguing they stay mad at each other). Each item was answered on a scale ranging from 1 (totally disagree) to 4 (fully agree), with higher scores indicating the students’ perception of the higher conflict frequency, the higher conflict intensity, and the worse the conflict resolution effect. Several reversed items (e.g., I never see my parents arguing or disagreeing) were recoded for all analyses. Previous studies have confirmed the reliability and validity of the scale (Li and Ye, 2024; Nigg et al., 2009). Cronbach’s *α* in the present study of each dimension and the total scale were 0.77, 0.80, 0.80, and 0.91, respectively.

#### 2.2.3 Shyness

Shyness was measured using the 13-item adapted Chinese version of the Shyness Scale (Cheek and Buss, 1981), which has a one-factor structure. Each item (e.g., I am socially somewhat awkward) was answered on a scale ranging from 1 (strongly disagree) to 5 (strongly agree). One reversed item (i.e., I do not find it hard to talk to strangers) was recoded for all analyses. We used the mean score for all the scale items as a total score, with higher scores indicating a higher level of shyness. This scale has shown good psychometric properties in previous studies (Hopko et al., 2005; Sato et al., 2018). Cronbach’s *α* of the total scale in the present study was 0.79.

#### 2.2.4 Loneliness

Loneliness was measured using the two-item adapted Chinese version of the Emotional versus Social Loneliness Scale (Russell et al., 1984). The two items were developed to assess students’ experiences of social and emotional loneliness (i.e., A possible type of loneliness involves not belonging to a group or social network. While this may be a set of friends who engage in social activities together, it can be any group that provides a feeling of belonging based on shared concerns, work or other activities. A possible type of loneliness is the lack of an intense, relatively enduring relationship with one other person. While this relationship is often romantic, it can be any one-to-one relationship that provides feelings of affection and security.). Each item was answered on a scale ranging from 1 (not at all) to 9 (very severely), with a higher score representing a higher level of loneliness. We used the mean score for all the scale items as a total score. This scale has shown good psychometric properties in previous studies (e.g., Wang et al., 1999). Cronbach’s α in the present study of the total scale was 0.75.

### 2.3 Data analysis

SPSS 23.0 was used for data entry and management; SPSS 23.0 and AMOS 17.0 were used for data analysis.

## 3 Results

### 3.1 Descriptive statistics

In terms of parental conflict, the perceived level of parental conflict among adolescents was generally at a low level (*M_parental conflict_* = 1.03), and the difference between genders was not significant, but the difference between grades was significant. Specifically, the scores of the second-grade students were significantly higher than those of the first-grade students, while the differences between the second grade and the third grade or between the third grade and the first grade were not significant [*F_(2, 1,037)_* = 3.31, *p* < 0.05; *M_second grade_*-*M_first grade_* = 0.67, *p* < 0.05; *M_second grade_*-*M_third grade_* = 0.12, *p* > 0.05; *M_third grade_*-*M_first grade_* = 0.54, *p* > 0.05].

In terms of socially adverse emotions, the shyness and loneliness of adolescents were generally at the lower-middle level (*M_shyness_* = 2.61, *M_loneliness_* = 3.93), and there were significant gender and grade differences. Specifically, boys were more shy than girls (*M_boys_* = 34.83, *M_girls_* = 33.18, *t* = 3.16, *p* < 0.01) and felt more lonely (*M_boys_* = 8.14, *M_girls_* = 7.60, *t* = 2.01, *p* < 0.05). In terms of shyness, there was no significant grade difference [*F_(2, 1,038)_* = 1.25, *p* > 0.05]. In terms of loneliness, compared with the first grade, the second grade experience more loneliness, while the difference between the first grade and the third grade, the second grade and the third grade was not significant [*F_(2, 1,038)_* = 4.05, *p* < 0.05; *M_second grade_*-*M_first grade_* = 0.80, *p* < 0.05; *M_third grade_*-*M_first grade_* = 0.54, *p* > 0.05; *M_second grade_*-*M_third grade_* = 0.27, *p* > 0.05].

In terms of family functioning, the family closeness felt by teenagers was generally at the upper-middle level (*M_Closeness_* = 4.31), and the difference between grades was not significant, but the difference between genders was significant. Specifically, girls felt significantly higher family closeness than boys (*M_boys_* = 68.03, *M_girls_* = 69.85, *t* = −2.46, *p* < 0.01). Teenagers felt family adaptability in general at the upper-middle level (*M_adaptability_* = 3.39), and there were no significant differences between gender and grade. Table 1 shows the detailed results.

**Table 1:** Descriptive statistics.

Variable	Overall (*M* ± *SD*)	Boys (*M* ± *SD*)	Girls (*M* ± *SD*)	t	Seventh grade (*M* ± *SD*)	Eighth grade (*M* ± *SD*)	Ninth grade (*M* ± *SD*)	F
Family functioning								
Closeness	69.00 ± 11.90	68.03 ± 11.00	69.85 ± 12.57	−2.46^**^	69.29 ± 11.99	68.40 ± 11.75	69.95 ± 12.04	1.09
Adaptability	47.45 ± 11.12	46.96 ± 10.59	47.88 ± 11.55	−1.32	47.32 ± 11.09	47.05 ± 10.81	49.37 ± 12.15	2.17
Parents conflict								
Frequency	13.80 ± 4.09	13.86 ± 3.91	13.75 ± 4.24	0.44	13.54 ± 4.24	13.92 ± 3.85	14.33 ± 4.23	2.23
Intensity	15.36 ± 4.79	15.49 ± 4.64	15.25 ± 4.92	0.80	15.12 ± 4.90	15.44 ± 4.67	16.01 ± 4.75	1.80
Resolution	10.99 ± 4.01	10.94 ± 3.84	11.03 ± 4.16	−0.36	10.64 ± 4.03	11.31 ± 3.91	11.19 ± 4.21	3.31^*^
Socially adverse emotions								
Shyness	33.94 ± 8.42	34.83 ± 7.92	33.18 ± 8.75	3.16^**^	34.01 ± 8.07	34.18 ± 8.07	32.85 ± 10.59	1.25
Loneliness	7.85 ± 4.31	8.14 ± 4.30	7.60 ± 4.31	2.01^*^	7.45 ± 4.28	8.26 ± 4.26	7.99 ± 4.47	4.05^*^

^*^p < 0.05, ^**^p < 0.01, ^***^p < 0.001.

### 3.2 Correlational statistics

The results of the correlational analysis (see Table 2) showed that each dimension of parental conflict was significantly negatively correlated with each dimension of family functioning and significantly positively correlated with adolescents’ shyness and loneliness. This indicates that the more frequent and intense the conflict, the poorer the degree of conflict resolution, the lower the family cohesion and adaptability, and the higher the likelihood that teenagers will experience socially adverse emotions. At the same time, family closeness and adaptability were significantly negatively correlated with adolescents’ shyness and loneliness, indicating that the better family functioning is, the less likely teenagers are to experience socially adverse emotions.

**Table 2:** Correlation matrices for study variables.

	1	2	3	4	5	6	7
1. Closeness	1						
2. Adaptability	0.78^**^	1					
3. Frequency	−0.39^**^	−0.33^**^	1		.		
4. Intensity	−0.40^**^	−0.38^**^	0.76^**^	1			
5. Resolution	−0.41^**^	−0.33^**^	0.57^**^	0.61^**^	1		
6. Shyness	−0.15^**^	−0.13^**^	0.18^**^	0.16^**^	0.15^*^	1	
7. Loneliness	−0.29^**^	−0.26^**^	0.16^**^	0.18^**^	0.21^**^	0.23^**^	1

^*^p < 0.05, ^**^p < 0.01.

### 3.3 Test of the mediation model

**Figure 2:**
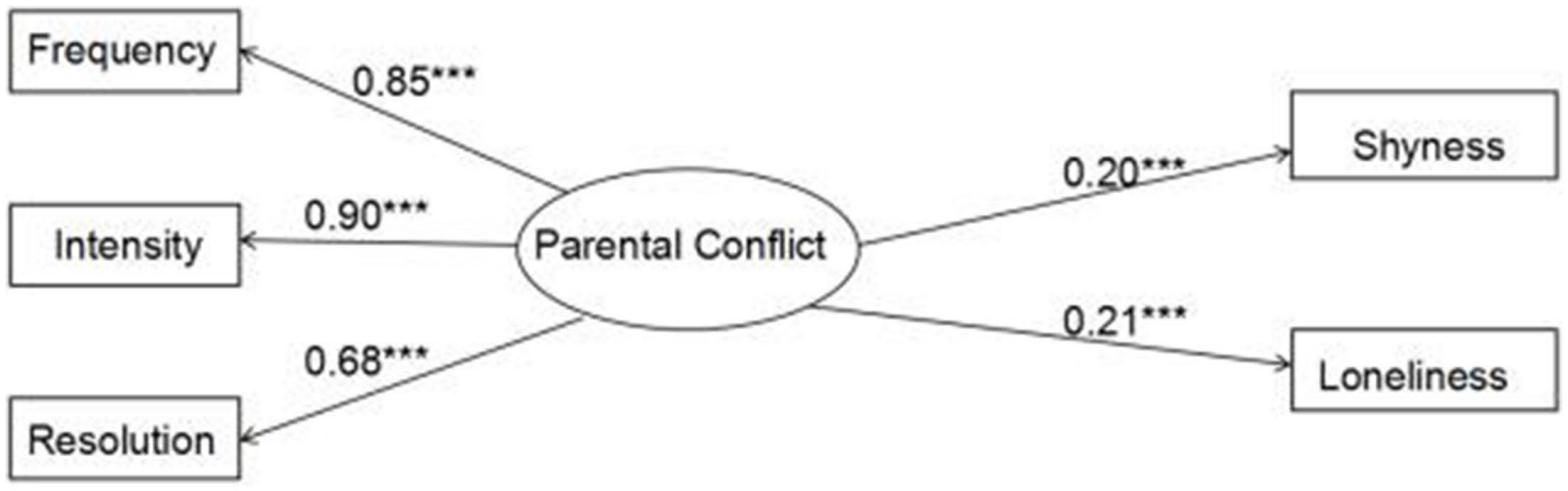
Impact model of parental conflict on adolescents’ socially adverse emotions.

First, the predictive effect of parental conflict on adolescents’ socially adverse emotions was detected. The results showed that *χ*^2^/df = 10.475, RMSEA = 0.096, NFI = 0.966, TLI = 0.939, CFI = 0.969, indicating that all the fitting indicators of the model reached relatively good levels. Based on the model results (see Figure 2), parental conflict had significant positive predictive effects on adolescents’ shyness (*β* = 0.200, *p* < 0.001) and loneliness experience (*β* = 0.212, *p* < 0.001).

**Figure 3:**
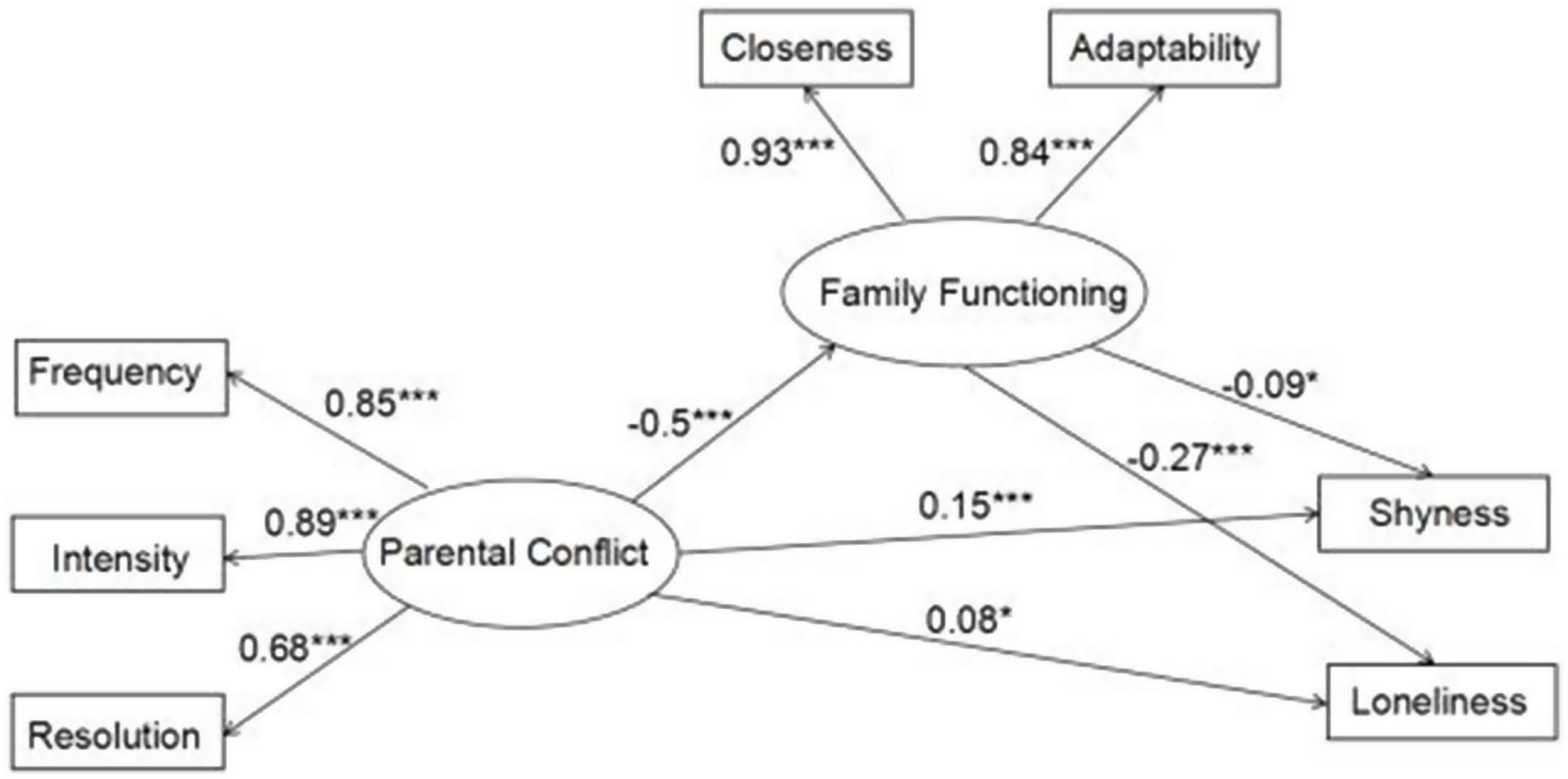
Validation model of the mediation effect of family functioning.

Next, a structural equation model was created by using family functioning as a mediating variable to verify the fitting degree of the hypothesized mediation model. The results showed that *χ*^2^/df = 7.380, RMSEA = 0.078, GFI = 0.978, NFI = 0.971, and CFI = 0.975 all reached satisfactory levels (see Figure 3).

As shown in Figure 3, after family functioning, the mediating variable was added, the path coefficient from parental conflict to adolescents’ shyness was still significant (*β* = 0.150, *p* < 0.001), and parental conflict significantly negatively predicted family functioning (*β* = −503, *p* < 0.001), family functioning significantly negatively predicted adolescents’ shyness (*β* = −0.092, *p* < 0.05); when the mediating variable of family functioning was added, the path coefficient between parental conflict and adolescents’ loneliness was still significant (*β* = 0.075, *p* < 0.05), parental conflict significantly negatively predicted family functioning (*β* = −0.503, *p* < 0.001), and family functioning significantly negatively predicted adolescent’ loneliness (*β* = −0.272, *p* < 0.001).

The mediating effect test was performed using the bootstrap method with a 95% confidence interval. The sampling was repeated 5,000 times. The mediation effect was significant if the confidence interval did not contain 0. This study reached two significant mediation chains: parental conflict → family functioning → adolescents’ shyness and parental conflict → family functioning → adolescents’ loneliness. The mediating effect values were 0.05 and 0.14, respectively (see Table 3). Since the direct effect of the first mediation chain is still significant, the mediation is regarded as partial mediation. Since the direct effect of the second mediation chain is not significant, therefore, the mediation is complete. The results indicated that family functioning played a partial mediating role in the relationship between parental conflict and adolescents’ shyness and a complete mediating role in the relationship between parental conflict and adolescents’ loneliness.

**Table 3:** Results of the mediating effect test.

Modal pathways	Mediating effect	95% CI	
		Lower	Upper
PC FF AS	0.05^**^	0.01	0.09
PC FF AT	0.14	0.09	0.18

PC, Parental conflict; FF, Family functioning; AS, Adolescents’ shyness; AT, Adolescents’ loneliness. ^**^p < 0.01.

## 4 Discussion

### 4.1 The particularity of the second grade in junior high school

In this study, the perceived parental conflict, the experience of shyness, and the experience of loneliness in the second grade in junior high school were higher than those in the first and third grades. This is consistent with the views of “problematic second grade in junior high school” and “key second grade in junior high school.” The particularity of the second grade in junior high school can be explained by the teenagers themselves and their parents.

From the perspective of the developmental characteristics of teenagers, those in the second grade of junior high school, who are 13 or 14 years old, are in the “storm period” of physical and mental development. At the level of psychological development, the pursuit of autonomy was the most important theme in this period. The pursuit of autonomy means the rapid development of independence and self-awareness of teenagers. On the one hand, this is manifested in rebellion at the behavioral level and the attempt to show their individuality; on the other hand, it is manifested in excessive self-focus at the conscious level, caring very much about others’ evaluations of themselves, and even many sometimes exaggerating the degree of attention others pay to them. This rebellion and self-focus, which may reach its peak in the second grade of junior high school, will cause friction or conflict with the outside world, causing teenagers to experience more loneliness. At the same time, individuals in the rebellious or self-focused state are prone to be irritated by little things and are prone to mistake subtle frictions for conflicts. Therefore, conflicts between their parents will increase to a higher level than the conflicts’ actual level.

In terms of physiological changes, male and female middle school students who entered adolescence began to show physiological changes, with the biggest changes among 13- and 14-year-old junior high school students. As sexual development begins to mature, teenagers gradually close themselves to the outside world. At the same time, their emotions toward the opposite sex gradually develop along with their physical and psychological development. They begin to have a hazy affection for the opposite sex. Focusing on the impression one has in the eyes of friends and the image among classmates of the opposite sex may also be an important reason why the second year of junior high school became a turning point for adolescents’ shy behavior and shy cognition.

From the perspective of parents, because the first grade of junior high school has just been promoted from elementary school to junior high school, the new environment and the new peer relationship has not yet been established, there are many problems in life, study, and psychological adaptation that require parents’ help and support. The third grade of junior high school is the top priority for many Chinese families. At these critical moments, to allow their children to adapt to junior high school life or prepare for the high school entrance examination with peace of mind, parents will consciously reduce the frequency of conflicts between them or reduce the intensity of conflicts if they cannot be avoided. In contrast, the second grade of junior high school is in the middle stage, and students have relatively fewer practical difficulties and pressures. Therefore, parents may not deliberately avoid conflicts, so teenagers in the second grade of junior high school perceive more parental conflict. According to the individual-environment interaction model, internalizing issues such as individual emotions will be affected by factors such as the family environment. The more frequent and severe the children’s perceived parental conflict, the more unfavorable the child’s healthy emotional development, which explained why the levels of shyness and loneliness in the second grade were higher than those in the first and third grades.

### 4.2 Discussion of gender differences

In terms of family functioning, girls reported significantly higher family cohesion than boys; in terms of socially adverse emotions, their experiences of shyness and loneliness were significantly lower than those of boys. Traditionally, girls were sensitive and fragile, while boys were dominant and competitive. Under the influence of this traditional concept, parents generally give girls more help and attention in life and give boys more restrictions and control, which to a certain extent makes girls perceive more closeness between family members than boys. Second, during adolescence, influenced by gender stereotypes, boys tend to take a tough stance and show their aggression in their dealings with others, while girls are encouraged to be friendly, cooperative, and value their relationships with others. Therefore, girls pay more attention to close relationships with family and show more pro-social behaviors, while boys have difficulty actively expressing their inner emotional distress and experience more shyness and loneliness.

### 4.3 Relationship between parental conflict, family functioning, and adolescents’ socially adverse emotions

From the results of the correlation analysis, it can be seen that the higher the level of parental conflict (i.e., the higher the intensity, the higher the frequency, and the poorer the conflict resolution), the higher the adolescents’ experience of shyness and loneliness. This validated the hypothesis of the present study and was consistent with previous studies (Hastings et al., 2015). Cognitive-situation theory can well explain the relationship between parental conflict and adolescents’ shyness. According to this theory, parental conflict makes adolescents feel threatened and even self-blame (Jordan, 2016). In the face of parental conflict, children tend to blame themselves for the discord between their parents, believing that the parent’s conflict is caused by themselves, and their self-evaluation will be reduced (He et al., 2023). Low self-evaluation can lead to children’s fear of others’ negative evaluation of them in the social process, and this is precisely the reason why children show shyness in social interactions. From the definition of loneliness, the reason why children feel lonely and are not accepted by their peers is because their behavioral problems such as aggression and withdrawal, as well as a lack of social interaction ability. According to Bandura’s social learning theory, parental conflict occurs either through the cold war or through quarrels, physical friction, or the way in which one party quarrels and the other party avoids or accommodates. Children who grow up in this environment for a long time will learn this kind of behavior and will be rejected by their peers and feel isolated and lonely.

Additionally, in the present study, the higher the level of parental conflict was, the lower the adolescents’ reported levels of family cohesion and adaptability. This was generally consistent with previous studies (Deng and Li, 2024). Family is a system, with the relationship between husband and wife as an important part of it. Frequent or high-intensity conflicts will not only destroy the emotional bond between two people but also break the harmonious atmosphere of the whole family and indirectly destroy the emotional bond between family members. In turn, the closeness between family members will be affected, and the family’s resilience will be reduced. In addition, many empirical studies have also confirmed that in families with high levels of parental conflict, children are more likely to be involved in a parent–child triangle relationship. In these relationships, children replace their parents in taking care of the family, while parents are more likely to beat and scold their children, forcing the children to become the “scapegoat” in the conflict between husband and wife. It is more likely that these kids will form an alliance with one parent against the other (Harman et al., 2022). These relationships confuse family roles and task division among family members, weaken the ability of the family to solve problems, and cause family functioning to begin to malfunction.

### 4.4 Mechanisms by which family functioning affects the relationship between parental conflict and adolescents’ social emotions

The results of structural equation modeling showed that the relationship between parental conflict and adolescents’ social impairment was mediated by family functioning, which was consistent with the research hypothesis and well supported the proximal-distal theory (Kroencke et al., 2023; Lionetti et al., 2024). An individual’s psychological development is the result of the continuous interaction between them and their surrounding environment; the distal factors in the ecosystem will affect the development of the individual through the proximal environment. Compared with parental conflict, which only reflects the relationship between parents, for adolescents, family functioning is the proximal environment that affects adolescents’ psychological development.

The results of the bootstrap analysis showed that family functioning played different mediating roles between parental conflict and adolescents’ shyness and loneliness. On the one hand, family functioning completely mediated the relationship between parental conflict and adolescents’ loneliness, i.e., after the family functioning factor was added, the direct effect of parental conflict on adolescents’ loneliness was no longer significant; on the other hand, parents’ family functioning played a partial mediating role in the relationship between conflict and adolescents’ shyness. That is, after the family functioning factor was included, the direct effect of parental conflict on adolescents’ shyness was still significant. As two important indicators of social distress in teenagers, why do family functioning have different effects on loneliness and shyness? This may be because compared to shyness, loneliness is more closely related to family functioning. Family and peers are two very important environmental factors that affect an individual’s loneliness. During adolescence, children have not yet been separated from their parents and the family is their main living environment, so the influence of family functioning, especially family closeness, on their loneliness becomes more important.

According to previous studies, there are many reasons for shyness. Parents’ neglect and indifference, individual poor attribution style and coping style, and unreasonable self-protection mechanisms are all important predictors of adolescents’ shyness. Family factors are only some of the factors. The results of the correlation analysis in this study also showed that the correlation coefficient between loneliness and each dimension of family functioning was significantly higher than that between shyness and each dimension of family functioning [*t_(closeness)_* = 3.808, *p* < 0.001; *t_(adaptability)_* = 3.499, *p* < 0.001]. However, in general, good family functioning has a positive effect on adolescents’ socially adverse emotions.

## 5 Implications and limitations

This study has important practical implications. First, parents must pay attention to the negative effect of parental conflict on adolescents’ socially adverse emotions (shyness, loneliness) and try their best to reduce parental conflict. Even if some conflicts are unavoidable, try to avoid them happening in front of your children. Second, when conflicts occur between parents, try not to let these conflicts affect parent–child relationship as well as the adaptability and problem-solving ability of the whole family when faced with problems.

There are several limitations to this study that should be acknowledged. With the advent of the internet, virtual spaces have become available for people to interact, leading to numerous studies exploring the relationship between loneliness, shyness and social media use (Appel and Gnambs, 2019; Wang et al., 2024). Does social media alleviate or exacerbate adolescents’ socially adverse emotions? Past research has shown mixed results (Gil de Zuniga et al., 2017; Wang et al., 2024). Additionally, previous research has indicated that the link between social media use and loneliness can vary depending on one’s shy tendency. However, the researchers’ results are inconsistent whether social media users with shyness tendency experience lower levels of loneliness (Gil de Zuniga et al., 2017) or higher levels of loneliness (Frison and Eggermont, 2015) as their social media usage time lengthens. Therefore, future research on adolescents’ shyness and loneliness might explore the extent to which and how an adolescent’s use of social media impacts them. The second limitation refers to the sample that cannot be considered representative of adolescents in China, thus limiting the generalizability of the findings. Moreover, because this study is cross-sectional, it cannot establish causality between variables. Future studies may include longitudinal research to expand the understanding of the nature of the relationships between these variables.

## Data Availability

The raw data supporting the conclusions of this article will be made available by the authors, without undue reservation.
